# Predictors of adverse events after endovascular abdominal aortic aneurysm repair: A meta-analysis of case reports

**DOI:** 10.1186/1752-1947-2-317

**Published:** 2008-09-30

**Authors:** Felix JV Schlösser, Geert JMG van der Heijden, Yolanda van der Graaf, Frans L Moll, Hence JM Verhagen

**Affiliations:** 1 Julius Center for Health Sciences and Primary Care, University Medical Center Utrecht, Utrecht, The Netherlands; 2 Department of Vascular Surgery, University Medical Center Utrecht, Utrecht, The Netherlands; 3 Department of Vascular Surgery, Erasmus Medical Center, Rotterdam, The Netherlands

## Abstract

**Introduction:**

Endovascular abdominal aortic aneurysm repair is a life-saving intervention. Nevertheless, complications have a major impact. We review the evidence from case reports for risk factors of complications after endovascular abdominal aortic aneurysm repair.

**Case presentation:**

We selected case reports from PubMed reporting original data on adverse events after endovascular abdominal aortic aneurysm repair. Extracted risk factors were: age, sex, aneurysm diameter, comorbidities, re-interventions, at least one follow-up visit being missed or refusal of a re-intervention by the patient. Extracted outcomes were: death, rupture and (non-)device-related complications.

In total 113 relevant articles were selected. These reported on 173 patients. A fatal outcome was reported in 15% (*N *= 26) of which 50% came after an aneurysm rupture (*N *= 13). Non-fatal aneurysm rupture occurred in 15% (*N *= 25). Endoleaks were reported in 52% of the patients (*N *= 90). In half of the patients with a rupture no prior endoleak was discovered during follow-up. In 83% of the patients one or more re-interventions were performed (*N *= 143). Mortality was higher among women (risk ratio 2.9; 95% confidence interval 1.4 to 6.0), while the presence of comorbidities was strongly associated with both ruptures (risk ratio 1.6; 95% confidence interval 0.9 to 2.9) and mortality (risk ratio 2.1; 95% confidence interval 1.0 to 4.7). Missing one or more follow-up visits (≥1) or refusal of a re-intervention by the patient was strongly related to both ruptures (risk ratio 4.7; 95% confidence interval 3.1 to 7.0) and mortality (risk ratio 3.8; 95% confidence interval 1.7 to 8.3).

**Conclusion:**

Female gender, the presence of comorbidities and at least one follow-up visit being missed or refusal of a re-intervention by the patient appear to increase the risk for mortality after endovascular abdominal aortic aneurysm repair. Larger aneurysm diameter, higher age and multimorbidity at the time of surgery appear to increase the risk for rupture and other complications after endovascular abdominal aortic aneurysm repair. These risk factors deserve further attention in future studies.

## Introduction

Up to the last decade of the last century, open surgery was the procedure of choice for abdominal aortic aneurysm (AAA) repair. Today, however, a minimally invasive endovascular procedure can be performed. Randomised trials show that short-term survival is better after endovascular abdominal aortic aneurysm repair (EVAR) than after open AAA repair [1,2]. After 2 years of follow-up, the total cumulative mortality in both groups is the same owing to excess mortality in the endovascularly treated group [3,4]. Randomised trials provide generally good evidence of causal effects of treatments, but the quality of evidence on the risk of adverse events is less satisfactory. This may often be the result of the selection of relatively healthy patients and the limited length of follow-up.

Extensive and long-lasting follow-up screening is generally required after EVAR. These extensive follow-up examinations may be a considerable burden for patients and health care providers, but they are necessary for early detection of postoperative complications [5,6]. Most complications are graft related and include graft migration, endoleak, graft thrombosis and AAA rupture. Rehospitalisation and re-intervention is necessary to treat many of these complications. Two European registries have reported a 3% risk of complications per year and a 10% risk of re-interventions per year [7-9]. Counterintuitively, registry data have shown that the risk of complications is significantly lower in patients who missed at least one follow-up visit compared with patients who attended all visits [10]. It is likely that these results are the consequence of selective surveillance in patients who are at increased risk for complications. Currently, no agreement exists on the optimal post-procedural surveillance regimen and the impact of frequent follow-up visits on the risk of complications after EVAR [11-13].

Evidence regarding the risk of complications after EVAR and predictors of these risks is lacking. Better insight into risk factors for complications after EVAR may lead to improvements in the efficiency of follow-up and patient selection. The aim of this study is to provide more insight into determinants of prognosis after EVAR by unique means: a meta-analysis of case reports.

### Data sources and study selection

The PubMed-Medline database was searched for case reports published up to January 2006. The following search string was used: ((('aorta' *and *'aneurysm') *or *('Aortic Aneurysms, Abdominal' [MESH])) *and *'endovascular' *and *'Case Reports' [pt]).

Titles, abstracts and full-text publications were obtained and screened for original data on adverse events after EVAR. Exclusion criteria were: 1, non-abdominal aneurysm; 2, inflammatory abdominal aortic aneurysm; 3, AAA rupture treatment. No language restrictions were applied. Full-text versions were obtained of all remaining articles.

### Data extraction and quality assessment

The following data about risk factors were extracted from the selected articles: age, gender, AAA diameter, comorbidities, endograft brand and type, one or more follow-up visits being missed and refusal of a re-intervention by the patient. The following data about clinical endpoints were documented: death, device-related complications and non-device-related complications. When a patient experienced more than one complication, all complications were documented. Device-related complications included: AAA rupture, endoleak types I, II, III, IV and V (endotension), graft infection, graft migration, graft thrombosis, graft kinking, stent wire fracture and technical mal-deployment. Non-device-related complications included cardiac, pulmonary and renal complications, fistula, ischaemia, multiple organ failure and other non-device-related complications.

### Data synthesis and analysis

Risk factors were associated with clinical endpoints by cross-tabulation. Risk ratios (RRs) and 95% confidence intervals (CIs) were calculated using Episheet [14]. A P value of less than 0.05 was considered significant.

## Case presentation

The Medline search strategy resulted in a total of 353 case reports. After excluding articles on the basis of the inclusion and exclusion criteria, 113 case reports remained which reported original data about 173 patients who had undergone endovascular AAA repair.

Table 1 shows baseline characteristics of the study population. Eighty percent of the patients were male (*N *= 138), 14% female (*N *= 24) and no data were available about gender in 6.3% of the patients (*N *= 11). The mean AAA diameter prior to device implantation was 60 mm (standard deviation 11; range 42 to 95). The AAA diameter was smaller than 5.5 cm in 25% of all patients (*N *= 43). The mean age was 73 years (range: 52 years to 93 years).

**Table 1:** Characteristics of the study population

	*N *or mean ± standard deviation	Percentage or range
**Gender**		
Male	138	80%
Female	24	14%
Unspecified	11	6%
**Age at operation (years)**	72.47 ± 7.62	(52 to 93)
50 to 59 years	7	4%
60 to 69 years	41	24%
70 to 79 years	83	48%
80 to 89 years	26	15%
90 to 99 years	1	1%
Unspecified	15	9%
**Comorbidities**		
Diabetes	5	3%
Smoking	5	3%
Hypertension	21	12%
Hypercholesterolaemia	6	3%
Cardiac status	25	14%
Obesity	7	4%
Stroke	5	3%
Pulmonary status	21	12%
Renal status	10	6%
Other*	23	13%
Peripheral vascular disease	7	4%
Carotid disease	1	1%
**Number of comorbidities**		
0 or unspecified	114	66%
1 or 2	26	15%
≥3	33	19%
**AAA diameter**	59.78 ± 11.04	42 to 95
**Incomplete follow-up adherence**^†^	8	5%
**Time interval between EVAR and complication (months)**	13.73 ± 16.11	0 to 85
Perioperative, up to 24 hours	31	18%
Initial, up to 30 days post-operative	28	16%
Short term, 30 days to 6 months	15	9%
Early mid-term, 6 months to 2 1/2 years	62	36%
Late mid-term, 2 1/2 years to 5 years	23	13%
Long term, > 5 years	4	2%
Unspecified	10	6%

*Other comorbidities that were described in the case reports included: active hepatitis C, alcohol abuse, arteriocaval fistula, bilateral gunshot injury, chemoradiation, cholangitis, Crohn's disease, factor VII deficiency, degenerative joint disease of lumbar spine, hemicolectomy, 'hostile' abdomen, hyperthyreoidectomy, hypoplastic marrow, liver cirrhosis, lymphoma, multiple gastrointestinal and urogenital operations, non-Hodgkin's lymphoma, pancreatoduodenectomy, pancytopenia, polycystic kidney disease, prostate cancer, rectal cancer, sigmoid resection and renal transplantation. †'Incomplete follow-up adherence' is defined by the patient missing one or more follow-up visits or refusing a re-intervention. AAA, abdominal aortic aneurysm; EVAR, endovascular abdominal aortic aneurysm repair; SD, standard deviation.

The median time from device implantation to death, rupture or other complications was 8.5 months with a range of 0 to 85 months. Table 2 provides an overview of the reported complications in our study population. A fatal outcome was reported for 15% of all patients (*N *= 26). AAA rupture caused death in 50% of these patients (*N *= 13). Death was directly or indirectly related to EVAR in the other 50% (*N *= 13), which mostly occurred after complications of conversion to open AAA repair or aortoduodenal fistula.

**Table 2:** Complications after endovascular abdominal aortic aneurysm repair

Complication	*N*	Percentage
**Device related**		
Endoleak	98	57%
Type I	25	14%
Type II	26	15%
Type III	12	7%
Type IV	0	0%
Type V/endotension	5	3%
Unspecified	30	17%
Kinking of stent graft	9	5%
Thrombosis of stent graft	19	11%
Graft migration	26	15%
Stent wire fracture	12	7%
Graft infection	5	3%
Technical deployment problems	13	8%
**Non-device related**		
Multiple organ failure	8	5%
Cardiac	7	4%
Pulmonary	8	5%
Renal	8	5%
Fistula	11	6%
Ischaemic, embolic	25	14%
Other*	6	3%
**Secondary intervention**	144	83%
Open conversion	57	33%
**AAA rupture**	38	22%
**Fatal course**	26	15%

*Other complications that were described in the case reports included: heparin-induced thrombocytopenia, metal-induced pruriginous dermatitis, peri-aortitis with ureteral obstruction, upper gastric intestinal bleed, sloughing of scrotal skin and impotence. AAA, abdominal aortic aneurysm.

AAA rupture occurred in 22% of all patients (*N *= 38). The AAA rupture was fatal in 34% of these patients (*N *= 13) and non-fatal in 66% of these patients (*N *= 25). Interestingly, in 50% of the patients with an AAA rupture (*N *= 19), no prior endoleak was detected during regular postoperative follow-up. Other complications that were reported for patients in the total study population included endoleaks in 52%, graft thrombosis in 11% and graft infections in 3%. Technical device-related complications, including mal-deployment of the graft, graft migration, graft kinking and stent wire fracture, occurred in 35% of all patients (*N *= 61). Non-device-related complications occurred in 42% of all patients (*N *= 73).

One or more re-interventions were performed in 83% of all patients. The main indications for re-intervention included embolisation, conversion to open AAA repair, clipping of arteries, operative exploration, thrombectomy and thrombolysis. Table 3 shows the calculated RRs and 95% CIs of associations of clinically relevant factors with subsequent mortality and rupture after EVAR. The risk of mortality was higher for female patients than for male patients (RR 2.9, 95% CI 1.4 to 6.0). A patient missing one or more follow-up visits or refusing a re-intervention appeared to increase the risk of both rupture and mortality (RR 4.7, 95% CI 3.1 to 7.0; and RR 3.8, 95% CI 1.7 to 8.3, respectively). The presence of at least three comorbidities was also significantly associated with rupture and mortality (RR 1.6, 95% CI 0.9 to 2.9; and RR 2.1, 95% CI 1.0 to 4.7, respectively). Larger AAA diameter and higher age appeared to be associated with increased AAA rupture risks, although none of the associations reached significance.

**Table 3:** Risk ratios and 95% confidence intervals of associations of clinically relevant factors with subsequent mortality and rupture after endovascular abdominal aortic aneurysm repair

		Death or rupture			Rupture			Death		
	***N *total**	***N *events**	**Risk**	**RR (95%CI)**	***N *events**	**Risk**	**RR (95%CI)**	***N *events**	**Risk**	**RR (95%CI)**

**Gender**										
Male	138	36	0.26	-	29	0.21	-	16	0.12	-
Female	24	11	0.46	1.8 (1.0;2.9)*	6	0.25	1.2 (0.6;2.6)	8	0.33	2.9 (1.4;6.0)*
Unspecified	11	4	0.36	1.4 (0.6;3.2)	3	0.27	1.3 (0.5;3.6)	2	0.18	1.6 (0.4;6.0)

**Age at operation **										

50 to 59 years	7	2	0.29	-	1	0.14	-	2	0.29	-
60 to 69 years	41	10	0.24	0.9 (0.2;3.0)	9	0.22	1.5 (0.2;10)	3	0.07	0.3 (0.1;1.3)
70 to 79 years	83	24	0.29	1.0 (0.3;3.4)	15	0.18	1.3 (0.2;8.2)	12	0.14	0.5 (0.1;1.8)
80 to 89 years	26	10	0.38	1.3 (0.4;4.8)	9	0.35	2.4 (0.4;16)	6	0.23	0.8 (0.2;3.2)
90 to 99 years	1	1	1.00	3.5 (1.1;11)*	1	1.00	7.0 (1.1;43)*	1	1.00	3.5 (1.1;11)
Unspecified	15	4	0.27	0.9 (0.2;3.9)	3	0.20	1.4 (0.2;11)	2	0.13	0.5 (0.1;2.7)

***N *comorbidities**										

0 or unspecified	114	27	0.24	-	24	0.21	-	13	0.11	-
1 or 2	26	8	0.31	1.3 (0.7;2.5)	3	0.12	0.5 (0.2;1.7)	5	0.19	1.7 (0.7;4.3)
≥3	33	16	0.48	2.0 (1.3;3.3)*	11	0.33	1.6 (0.9;2.9)	8	0.24	2.1 (1.0;4.7)*

**AAA diameter**										

40 to 49 mm	15	5	0.33	-	3	0.20	-	3	0.20	-
50 to 59 mm	67	19	0.28	0.9 (0.4;1.9)	13	0.19	1.0 (0.3;3.0)	11	0.16	0.8 (0.3;2.6)
60 to 69 mm	36	10	0.28	0.8 (0.3;2.0)	8	0.22	1.1 (0.3;3.6)	5	0.14	0.7 (0.2;2.5)
70 to 79 mm	14	8	0.57	1.7 (0.7;4.0)	7	0.50	2.5 (0.8;7.8)	2	0.14	0.7 (0.1;3.7)
> 80 mm	11	4	0.36	1.1 (0.4;3.1)	3	0.27	1.4 (0.3;5.5)	3	0.27	1.4 (0.3;5.5)
Unspecified	30	5	0.17	0.5 (0.2;1.5)	4	0.13	0.7 (0.2;2.6)	2	0.07	0.3 (0.1;1.8)

AAA, abdominal aortic aneurysm; CI, confidence interval; RR, risk ratio. *P value less than 0.05.

## Discussion

Female gender, comorbidities, missing one or more follow-up visits or refusal of a re-intervention by the patient appear to significantly increase the risk for mortality after EVAR. No prior endoleak was discovered during follow-up in 50% of the patients with an AAA rupture after EVAR. Larger aneurysm diameter, higher age and comorbidities may also increase the risk for AAA rupture after EVAR, although these associations could not be established significantly.

To the best of the authors' knowledge this is the first meta-analysis of case reports. Case reports do not provide strong causal evidence because they report only a small number of patients. Case reports can provide relevant information, notably on long-term complications in the realm of patients actually seen and treated in daily practice. Although they could be emphasising the bizarre [15], case reports are considered an important cornerstone for medical progress. This type of article can help to detect specific patterns of patient outcomes, particularly with regard to clinically important and rare adverse events and complications [16]. Case reports may therefore offer valuable information about the mechanisms of the development of complications.

The aim of our study was to review which patient, disease or procedural characteristics predict complications after EVAR. The selection of case reports about patients with complications after EVAR may have resulted in a cohort of patients who are at high risk for complications, irrespective of the device or the procedure. Therefore, one may question whether these extraordinary patients may have brought the complications to the device or procedure. Although patients who were included in this study may represent the odd and extraordinary cases, they clearly are patients who are seen in practice. For ethical considerations and reasons of efficiency, these odd and extraordinary cases are generally excluded from randomised trials and cohort studies. The risk factors derived from the presented cohort of case reports are similar to those reported in prognostic cohort studies. Hence, our results contribute to the robustness of the reported predictors.

Unfortunately, the documentation of clinical data was not performed according to a standardised protocol [17] in many case reports. As data in our study were limited to data that were presented in the selected case reports, a considerable amount of data was missing. The percentages of missing data in our study were 6.3% for gender, 8.7% for age, 5.8% for the time interval between EVAR and complication, and 17% for initial AAA diameters. Univariate analyses were performed to calculate associations between putative risk factors and subsequent clinical outcomes for different subgroups on the basis of the available data and also for the group of patients with missing and/or unspecified data. Comorbidities were described in 34% of all patients. From our point of view, this percentage can best be regarded as the minimum value of the number of patients with comorbidities, because under-reporting of comorbidities is likely in the other 66%. Missing data is a disadvantage which is inevitably linked with the unique approach, and should be regarded carefully when interpreting the results.

Several studies have compared mortality and morbidity risks in men and women after EVAR. Two national database studies in the US have shown that mortality after EVAR is significantly 2.0 to 2.5 times higher in women than in men [18,19]. The EUROSTAR study indicated that female gender was a significant risk factor for endoleak [20]. In addition to significantly reduced sizes of iliacal arteries, women are more likely to have a shorter, more dilated and more angulated proximal aortic neck, which may lead to proximal endoleak and graft migration [21]. Female patients also have a higher risk of abortion of the initial EVAR procedure and mal-deployment of the endograft [22]. Wolf et al. showed that women had significantly more intra-operative complications compared with men. They hypothesised that this was related to differences in arterial access [23]. Nordness et al. showed that women were more likely to have significant arterial dissections during EVAR. One-month mortality risks were 12% in female and 0% in male patients (P = 0.02). One-month complication risks were 41% in women and 15% in men (P = 0.02) [24]. Ouriel et al. found no differences between men and women in perioperative and mid-term mortality. However, they demonstrated a higher risk for graft-limb occlusions in women than in men [25].

The impact of comorbidities on the risk of mortality after EVAR has been described by several authors. Azizzadeh et al. showed that patients with a low glomerular filtration rate (GFR) faired significantly worse than patients with a better GFR [26]. Biancari et al. showed that survival was significantly different among tertiles of the Glasgow Aneurysm Score, which is a tool for measuring the fitness of the patient for surgery (P < 0.001). Patients with a high score and extensive comorbidities had a significantly lower 5-year survival rate than the other patients [27]. Chaikof et al. categorised patients into a high-risk group (*N *= 123) and a low-risk group (*N *= 113) according to the clinical condition of the patient. The 2-year survival was 73.5% for high-risk patients and 85.8% for low-risk patients (P = 0.035 [28]. Riambau et al. showed that patients with a poor medical condition had a significantly lower 1-year survival after EVAR compared with relatively fit patients: 83% versus 93% (P < 0.001). Diabetes mellitus appears to influence mortality considerably [29]. Zannetti et al. divided patients in subgroups according to the American Society for Anesthesiology (ASA) classification. Cumulative survival was 89% in the ASA < IV and 76% in the ASA IV group (P = 0.004) after 3 years of follow-up [30]. These reports, in combination with our results, underscore the impact of comorbidities on mortality and morbidity after EVAR.

Missing one or more follow-up visit appeared to increase the risk of complications in our study. As far as we know, this has never been described before. The EUROSTAR study showed counter-intuitively that the risk of complications was significantly higher in patients with a perfect follow-up adherence. Compliance with follow-up screening in their study appeared to be biased, however, because high-risk patients, including smokers, patients with hyperlipidaemia, and patients who were unfit for open surgery or general anaesthesia had the best follow-up adherence [10]. Therefore, extensive follow-up screening and re-interventions are still required after EVAR.

## Conclusion

Although a meta-analysis of case reports has some clear methodological drawbacks, it offers unique opportunities. The risk factors for complications after endovascular AAA repair that are presented in this document are similar to those that are presented in prognostic cohort studies. Female gender and the presence of comorbidities appear to increase the risk of mortality after EVAR. Larger AAA diameter, higher age and multimorbidity at the time of surgery increase the risk for rupture and other complications following EVAR. These risk factors deserve attention in future well-designed follow-up studies.

## Abbreviations

AAA: abdominal aortic aneurysm; ASA: American Society for Anesthesiology; CI: confidence interval; EVAR: endovascular abdominal aortic aneurysm repair; GFR: glomerular filtration rate; RR: risk ratio; SD: standard deviation.

## Competing interests

The authors declare that they have no competing interests.

## Authors' contributions

Each author has participated sufficiently in the work to take public responsibility for appropriate portions of the content.
